# A Guide for the Inclusion of Sex and Gender Considerations in Clinical Practice Guidelines

**DOI:** 10.1002/gin2.70087

**Published:** 2026-09-11

**Authors:** Tania King, Jennifer Ervin, Marya Yenita Sitohang, Kim Dalziel, Michelle Tew, Cara Tannenbaum, Humaira Maheen

**Affiliations:** ^1^ Social Equity Research Centre, School of Global, Urban and Social Studies RMIT University Melbourne Australia; ^2^ Centre for Health Policy, Melbourne School of Population and Global Health University of Melbourne Melbourne Australia; ^3^ Faculty of Medicine Université de Montreal Montréal Québec Canada

## Abstract

Despite increasing understanding of sex and gender as important determinants of health, there has been a lack of consideration regarding sex, gender, and other intersectional factors in clinical practice guidelines (CPGs) to date, and little support for guideline developers to address these shortcomings. The development of a guide for guideline developers to facilitate the systematic uptake of sex and gender considerations may enhance the uptake and consistency of sex and gender considerations in CPGs. Informed by a comprehensive programme of work that included an umbrella review of the global literature, extensive expert consultation and input from individuals with living experience, the resultant guide provides practical step‐by‐step strategies for CPG developers to systematically integrate sex and gender considerations throughout the guideline development process. Aligning with key global guideline development steps, this guide addresses an important clinical need to translate sex and gender‐specific evidence into tailored clinical action, where relevant, and provides recommended pathways to improve guideline quality and equity. The integration of sex and gender into CPGs is a global imperative, and whilst acknowledging that guidelines are developed and disseminated differently across different countries and contexts, this guide has international relevance.

## Summary

- Sex and gender are key determinants of health.
- Historically, there has been inadequate attention to the inclusion of sex and gender considerations in clinical practice guidelines, with implications for the treatment outcomes of different patient groups.
- This guide was developed to support guideline developers in incorporating sex and gender considerations into clinical practice guidelines.
- To improve guideline quality, optimise patient outcomes, and support health and gender equity, sex and gender considerations need to be incorporated into clinical practice guidelines.

## 1 Background

This guide supports the systematic integration of sex and gender considerations throughout guideline development. Recommendations align with key global guideline development steps while flexibly accounting for local context and setting.

Sex and gender intersect with other important determinants of health and health equity, including age, sexual orientation, ethnicity, disability, socioeconomic position, and geographic location [1]. This guide does not replace comprehensive equity guidance related to these other determinants; guideline developers should use this guide in conjunction with other equity resources for comprehensive consideration of health equity [2, 3].

Sex and gender matter for clinical practice guidelines because biomedical research has historically relied on male cells, male animal models and male humans as the default, with females systematically under‐represented in preclinical research through to clinical trials and data analysis [4, 5, 6]. Significant gaps persist, and sex and gender considerations remain inadequately incorporated into CPGs internationally [7, 8, 9, 10, 11]. Incorporating sex and gender considerations enhances guideline quality, appropriateness and translation. Guidelines that do not account for evidence on sex and gender differences may provide recommendations that are less effective for some populations, potentially contributing to misdiagnosis, adverse treatment outcomes and the perpetuation of physical and mental health inequities [12, 13]. Integrating sex‐ and gender‐based analyses and considerations throughout guideline processes is essential to ensuring equitable, evidence‐informed, and inclusive healthcare.

**Terminology**

Terminology around sex and gender continues to shift and evolve [14], however some key terms relevant to this document are noted below.

- Sex is typically recorded at birth on the basis of biological and physiological characteristics, including chromosomes, hormones and anatomy (external genitalia and internal reproductive organs). Variations of sex characteristics exist, meaning that not all individuals fit binary male or female sex categories. Sex is known to influence physiology, disease trajectories, and pharmacological outcomes [15]. For example, males and females may exhibit distinct cardiovascular disease presentations, metabolic profiles, immune responses, mental health symptom manifestations, as well as medication pharmacodynamics and pharmacokinetics [16, 17]. These sex‐based differences can have implications for diagnostic criteria, treatment options, pharmacological dosing and disease monitoring approaches.
- Gender is socially constructed and is neither fixed nor binary. It encompasses gender identity (how an individual understands and experiences their own gender); gender expression (how a person expresses their gender); gender norms, roles, attributes and expectations; and the power structures between genders. Gender is known to shape health‐related behaviours, exposure to risk factors, healthcare‐seeking behaviours and access to care [1]. Gender considerations may be less directly relevant to some clinical outcomes and recommendations, but are particularly important when addressing mental health, health behaviours, preventive care, screening and monitoring approaches, treatment adherence, healthcare delivery and hormone therapies. Gender identity may include, but is not limited to, man, woman, non‐binary and other gender‐diverse identities.
- Intersex: describes individuals who are born with sexual characteristics that do not align with binary conceptualisations of sex. Intersex is a naturally occurring variation and is distinct from an individual's gender identity or being transgender. In some countries, intersex individuals are referred to as people having variations of sex characteristics [18, 19].

- Cisgender: For most people, gender aligns with the sex that was recorded at birth (i.e., most people who had female recorded at birth identify as women and most people who had male recorded at birth identify as men). This is referred to as cisgender. For some groups, particularly transgender and non‐binary people, these two constructs may not align. Importantly, these individuals often face discrimination and a lack of tailored health care due to insufficient medical workforce training and guidelines that are not inclusive.
- Transgender: an umbrella term referring to people whose gender identity and/or expression is different from the sex recorded at birth [14]. This includes transgender men (recorded as female at birth, identify as male), transgender women (recorded male at birth, identify as female), and some non‐binary people.
- Gender diverse: an inclusive term describing people whose gender identity or expression differs from binary gender norms. This includes non‐binary, genderfluid, agender and other identities beyond the gender binary.
- Sexual orientation: an umbrella term that encapsulates sexual identity, sexual and romantic attraction, and sexual behaviour. Self‐defined, it may change over time and contexts and is distinct from sex and gender. While this intersects with sex and gender, sexual orientation is not covered by this guide.

The evidence gap on sex‐related outcomes has reduced in recent years; however, gender considerations in clinical outcomes remain less common. While both sex and gender warrant consideration in CPGs, gender considerations may be limited by data gaps, and in many cases, there will be insufficient data, particularly for diverse gender‐populations. For many conditions, guideline developers may therefore find that prioritising sex‐based considerations represents a practical and evidence‐supported first step. More comprehensive attention to gender considerations should be incorporated as evidence, methodological approaches, and resources develop.

Evidence on sex and gender differences in health is constantly evolving, concomitant with growing societal understanding and awareness of these constructs. This underscores the ongoing need for regular re‐evaluation and updating to ensure that recommendations remain reflective of current knowledge and best practice.

## 2 Methods

Recommendations were developed through a multi‐component methodology including expert and lived experience consultation, participatory research, and two literature reviews, with full methodological details provided in the Supplementary Materials. In‐depth qualitative interviews were conducted with 31 participants drawn from diverse backgrounds, disciplines, and sex and gender perspectives, including individuals with lived experience of gender and sexual diversity and sex‐specific health conditions. Two international literature reviews were conducted: an umbrella review [20] synthesising existing research on the inclusion of sex and gender in clinical practice guidelines, and a targeted analysis of best‐practice guidelines identified through that review. Guide development drew on findings from these reviews and interview findings alongside established frameworks for health equity in guideline development, including the GIN‐McMaster Guideline Development health equity considerations [21], the Cochrane Equity Methods Group (PROGRESS‐Plus) [22, 23], the Grading of Recommendations Assessment, Development and Evaluation (GRADE) equity guidelines [24], and the Sex and Gender Equity in Research (SAGER) guidelines [12, 25]. Australian guidelines were also used, including the National Health and Medical Research Council (NHMRC)'s Guideline Standards [26], *Guidelines for Guidelines: Equity* [3], and *Statement on Gender, Variations of Sex Characteristics and Sexual Orientation in Health and Medical Research* [18]. Feedback on the draft guide was obtained through a staged consultation process involving health research funders, clinicians, guideline developers, and lived experience consultants. Ethics approval was obtained from the University of Melbourne Human Research Ethics Committee (#2025‐33040‐68447‐3).

## 3 What to Do

The recommendations in this guide are structured to align with globally recognised steps for developing CPGs (Table 1). Although terminology, sequencing, and procedural detail may vary across frameworks, the core sex and gender considerations remain broadly consistent.

**Table 1:** Steps in international CPG development frameworks.

Step	WHO handbook [27] equivalent	NICE process [28] equivalent	GIN–McMaster checklist [29] equivalent	NHMRC [26] equivalent
1: Establishing guidelines development group	Forming the guideline development group (GDG)	Forming the decision‐making committees	Topics 3, 4, 6: Group membership, group processes, consumer involvement	Establishing your guideline development group and advisory panels
2: Scoping the guideline	Scoping and formulating PICO questions	Scoping the guideline and developing review questions	Topics 5 & 8: Establishing scope and priority questions (PICO)	Scoping your guideline
3: Undertaking the evidence review	Evidence retrieval, appraisal and synthesis	Identifying and reviewing the evidence	Topics 10–12: Evidence search, summarising evidence, judging quality/certainty	Undertaking evidence review
4: Formulating guideline recommendations	Formulating and grading recommendations (GRADE)	Interpreting the evidence: evidence to recommendations	Topics 13–14: Developing recommendations, wording of recommendations	Guideline recommendations
5: Structuring the guideline	Producing and publishing the guideline	Writing the guideline and consensus process	Topic 15: Reporting and Peer Review	Structuring your guideline
6: Undertaking public consultation	External review	Validation process and public consultation	Topic 15: Public consultation	Undertaking public consultation
7: Disseminating, implementing and evaluating the guideline	Planning for dissemination, implementation and evaluation	Implementing and updating the guideline	Topics 16–18: Dissemination, implementation, evaluation and updating	Disseminating, implementing and evaluating your guideline

Structuring the recommendations against steps in this way is intended to enable developers to systematically integrate sex and gender considerations throughout the guideline development process. The prompts and reflective questions throughout the guide are intended to support consideration and discussion and are not prescriptive. Sex and gender consideration should be integrated across the development process alongside other core methodological considerations (similar to certainty of evidence and equity assessment). Guideline developers should apply those most relevant to their specific condition, target population, and context. To enable adaptation to different countries and contexts, particularly those where resources are limited or where political pressures restrict the translation of gender‐related scientific evidence to practice, the recommendations have been flexibly labelled as core (C) (minimum requirements for all guidelines); high‐impact (HI) (priority where resources permit); and context‐specific (CS) (recommended where relevant to the condition, population, or guideline scope). Supplementary resource materials accompanying this guide include case studies, practical ‘how‐to’ examples for each stage of guideline development, as well as a sex and gender considerations checklist aligned to each step.

Given that sex and gender are distinct constructs, this distinction is reflected throughout the recommendations that follow. Where a recommendation refers specifically to ‘gender’, ‘sex’ or ‘sex and gender’ together, this is intentional. Where a recommendation addresses both sex and gender, this is stated explicitly. Guideline developers are encouraged to read each recommendation in light of these distinctions and, where the primary focus is on one construct, to consider whether the other may also warrant attention in their specific context.

### 3.1 Step 1. Establishing Guideline Development Group and Advisory Panels

*Core recommendation: Ensure diverse and inclusive gender representation in the guideline development group*

The composition of guideline committees is crucial for developing inclusive CPGs [30]. Meaningful engagement with women and gender‐diverse populations, and their representation on guideline panels brings essential perspectives that shape clinical questions, scope definition, outcome prioritisation, interpretation of evidence and recommendations. Without this diversity, panels may inadvertently frame guidelines around default assumptions that do not reflect the experiences or needs of all patient populations, embedding bias from the earliest stages of development, and ultimately compromising care quality and patient outcomes [18, 26, 31].

Guideline development processes should:

- Aim for women to hold at least 40% of positions; where current representation is lower, document barriers and develop a plan to increase representation. (C)
- Include individuals with disciplinary expertise in sex and gender (e.g., a sex and gender champion [32]) on guideline development and advisory panels. (C)
- Where relevant to health condition or population that is the focus of the guideline, engage people with lived experience of sexual and gender diversity [7, 8] so that guideline development is informed by diverse perspectives. For fields where representation of women is low, guideline development groups could ensure representation through inclusion as lived experience experts. (CS)

**Example:** The European Society of Cardiology (ESC) Guideline on Cardiovascular Disease Prevention [33] included sex and gender experts in the development team and consequently provided a more comprehensive integration of sex and gender considerations, offering clear definitions, explaining their impact on health, and differentiating risk factors, diagnostic approaches, and clinical recommendations for women and men.

### 3.2 Step 2. Scoping the Guideline

*Core recommendation: Identify sex and gender issues relevant to the guideline*

In scoping the guideline, the guideline development group should explicitly consider how sex and gender may be relevant to the problem or intervention.

Guideline development processes should:

- Explicitly consider sex and gender when developing clinical questions and defining target populations for CPGs [34]. Consider how sex or gender may affect access, diagnosis, treatment adherence or other relevant outcomes. (C)
- Consult with diverse sex and gender communities and those with lived experience. (CS)
- Specify sex‐ and gender‐disaggregated terms a priori. (C)
- Conduct scoping to identify known and potential sex or gender differences. The following prompts are illustrative, and guideline developers should consider those most relevant to their specific condition, target population and context.
  ∘ Epidemiology and presentation: Are there known or potential sex differences in the prevalence, incidence, symptom presentation or clinical course of the condition? (C)
  ∘ Biological mechanisms: Are there known or potential biological, hormonal, immunological, or metabolic differences between sexes that could affect disease pathophysiology, treatment safety or effectiveness? (C)
  ∘ Access, diagnosis and treatment: How might gender‐related factors affect diagnosis, treatment or access to care, including delayed diagnosis or under‐ or over‐prescribing? Are there known or potential sex or gender differences in the sensitivity of diagnostic tests or responses to treatment/intervention, including efficacy, safety, adverse effects or pharmacokinetics? (C)
  ∘ Evidence gaps: Are males or females under‐represented in the evidence base? Have sex‐disaggregated analyses been conducted? Are gender‐diverse or intersex populations considered? Where relevant, consider how hormone therapy might affect progression of the disease or response to treatment (e.g., taken by transgender individuals or for treatment of menopause/perimenopause). (C)
  ∘ Implementation factors: How might sex influence guideline implementation (e.g., monitoring needs)? How might gender be related to implementation (e.g., healthcare‐seeking behaviours, patient‐clinician communication, care delivery setting, adherence)? How do different intersectional identities interact with sex and gender? (HI)
  ∘ Equity implications: Could recommendations inadvertently exacerbate inequities, create barriers, or have unintended consequences that differentially affect people based on sex or gender? Are there life stage factors (such as pregnancy) that might have clinical implications? (C)

**Example:** Women often present with different cardiovascular symptoms to men (e.g., fatigue, nausea rather than classic chest pain), which can lead to their delayed diagnosis and under‐treatment. Identification of these differences could inform guideline recommendations and lead to improved outcomes for females with cardiovascular disease [35].

### 3.3 Step 3. Undertaking the Evidence Review

*Core recommendation: Ensure appropriate evidence is sought, identified and considered* 1

The following prompts represent examples to assist guideline developers. They are illustrative rather than prescriptive, and developers should select and apply those most relevant to their specific guideline context.

#### 3.3.1 Search Strategy, Evidence Review and Data Extraction

When systematically reviewing the evidence, explicitly incorporate sex and gender throughout the process by:

- Including sex‐ and gender‐specific search terms in your search strategy. (C)
- Seeking studies that examine sex and gender differences, or that specifically examine a single sub‐group. (C)
- Extracting sex‐disaggregated outcomes and baseline characteristics. (C)
- Recording whether studies report sex‐specific analyses and results. (C)
- Noting the sex or gender distribution of study populations. (C)

Depending on the state of the science in the field under review, additional searches may be warranted. For example, using terms such as ‘male’ and ‘female,’ ‘women’ and ‘men’ or searches targeting single‐sex studies or gender‐related issues. A sex and gender expert within the guideline development group may help in determining when and how complementary searches should be developed. Validated search strategies, such as those developed by Song et al., [36] are suggested as a starting point to improve identification of sex‐specific diagnostic and treatment evidence and better inform recommendations across different clinical questions [34].

#### 3.3.2 Evidence Synthesis and Subgroup Analysis

Where data permit, and where there is evidence or biological plausibility for sex or gender differences:

- Conduct or extract sex‐ or gender‐disaggregated analyses for clinical presentation, diagnostic accuracy, treatment efficacy, adverse effects, toxicity, and safety outcomes. (C)
- When data permit, and biological plausibility exists for conducting sex or gender subgroup analyses, consider the following:
  ∘ Assess whether sex and gender‐specific subgroup analyses were pre‐specified in the original studies. Although post‐hoc analyses may be under‐powered to detect statistical differences, they may still illustrate important trends requiring further investigation.^2^ (C)
  ∘ Recognise that exploratory post‐hoc subgroup analyses can have an increased risk of spurious findings due to multiple comparisons. If analyses are not pre‐specified, frame them as exploratory and treat them as hypothesis‐generating (as opposed to hypothesis‐confirming). (C)
  ∘ Evaluate whether sample sizes are adequate. Small samples may produce imprecise estimates with wide confidence intervals; however, reporting them may prompt further study or future meta‐analyses. (HI)
  ∘ Consider the influence of publication bias: examine whether subgroup analyses were planned but not reported, as null or non‐significant findings by sex or gender are less likely to be published. Where individual participant data are available, assess whether sex‐disaggregated analyses could be conducted but were not. Document and report both significant and null findings from subgroup analyses, as the absence of reported differences should not be assumed to reflect a true lack of difference. (C)
  ∘ Avoid over‐interpreting findings, especially when not pre‐specified or when there are data limitations. The goal is to inform best practice within each sex or gender group, not necessarily to compare statistical differences between groups. This means emphasis should be placed on evidence to treat each group, not whether there are meaningful treatment differences between them. (C)
  ∘ Consult a biostatistician when uncertain about the appropriateness or interpretation of sex‐specific analyses. Where statistical power is lacking, expert opinions based on data or clinical trends may assist. (HI)

It is important to note that if sex‐disaggregated data are not reported, this represents a critical evidence gap, rather than confirmation that no differences exist. Sometimes there are no known sex or gender differences precisely because there has been no examination of differences. This represents an important knowledge gap rather than evidence of no difference. As good guideline practice states, evidence gaps should be highlighted as possible areas for further research (and published in the guideline) (an example of what guideline developers could do in this situation is provided in the resources section (Step 7) of the Supplementary files).

#### 3.3.3 Evidence Appraisal

In assessing the quality, relevance, and applicability of evidence, consider the following criteria [34, 37]:

- Representation and applicability: Consider whether males and females are adequately represented in the included studies. Are the study populations representative of the guideline's target population? Is evidence applicable to intersex and gender diverse populations? (C)
- Sex or gender specific bias: Do eligibility criteria restrict participation by sex or gender? Is there systematic exclusion or under‐representation of one sex or gender in the evidence base that could lead to bias? Consider use of available tools for assessment of the risk for sex‐specific bias in included reports [34]. (HI)
- Quality of sex or gender‐specific evidence: Is the quality of evidence comparable across sex and gender? If disaggregated analyses exist, are they adequately powered and conducted appropriately? (HI)
- Biological plausibility: Do observed sex or gender differences (or lack of differences) align with biological plausibility of the condition and mechanism of the intervention? Applicability across populations: How likely is it that the published research results are applicable to all sexes? Are they applicable to gender‐diverse people? How might gender‐related factors affect the implementation or outcomes in practice? (HI)
- Benefits and harms: How likely is it that sex‐ and gender‐related differences exist in the balance between anticipated benefits and harms? Are there any implications for pregnancy and reproductive health? (C)

#### 3.3.4 Evidence‐to‐Decision (EtD) Frameworks

When using EtD frameworks, explicitly structure judgments regarding sex and gender considerations:

- Assess whether available evidence shows that benefits, harms or adverse effects differ by sex or gender. (C)
- Consider whether feasibility, acceptability or resource implications differ by sex or gender. (HI)
- Consider whether recommendations benefit one sex or gender more than others. (HI)
- Identify potential harms or barriers for specific populations. (HI)
- Consider whether clinical criteria (diagnostic thresholds, treatment eligibility, intervention pathways) are applied consistently across sex and gender due to bias in clinical practice, rather than evidence‐based differences. (C)
- Consider whether social values and preferences might be represented in the evidence base and whether the guideline development process reflects the perspectives of people of diverse sexes and genders, especially those most affected by recommendations. (CS)
- Evaluate whether baseline risk for the condition or adverse outcomes vary by sex or gender, recognising that differences in baseline disease risk may shift the benefit‐harm balance, even when effects of treatment are similar across groups. (C)

Throughout the evidence review process:

- Ensure transparent reporting. (C)
- Clearly document the rationale for any certainty of evidence judgements related to sex and gender. This could include: downgrading of evidence quality due to limited representation of sex or gender subgroups; concerns about potentials for gender bias in the evidence base; limitations in availability or quality of sex‐disaggregated data; imprecision in sex or gender‐specific estimates due to small samples; generalisability when evidence comes from one sex or gender, but recommendations apply to the whole population. (C)

**Example:** Assessing sex‐specific bias: Assessments of the risk for sex‐specific bias in research reports can be evaluated using the participation to prevalence ratio (PPR), a metric that links the participation of each sex to the prevalence of the disease of interest in the population. A PPR > 0.8 is considered adequate or bias‐free enrolment [34].

### 3.4 Step 4. Formulating Guideline Recommendations

*Core recommendation*: *Consider the impact of guideline recommendations on under*‐*represented sex and gender populations*

This stage of guideline development provides the opportunity for developers to reflect on whether considerations assessed in Step 3 above warrant different recommendations according to sex and gender, or whether qualifying statements about the generalisability and applicability of recommendations may apply to specific subpopulations [24, 29]. Developers should carefully consider potential unintended consequences regarding access to treatment and healthcare when making sex‐specific recommendations (e.g. one sex inadvertently receiving later access to life saving treatment on the basis of differential risk thresholds or stricter eligibility criteria). The following considerations provide examples to assist in formulating recommendations, noting again that not all will be relevant to every CPG.

Structuring recommendations when sex or gender differences are identified:

- Provide separate recommendations for males and females when evidence shows clinically meaningful differences that align with biological plausibility in treatment effects, safety profiles, or outcomes (e.g., different drug doses, different diagnostic thresholds). (C)

- Adjust recommendation strength to reflect the quality and certainty of evidence for each sex:
  ∘ Use conditional/moderated recommendations when evidence is limited to or predominantly from one sex. (C)
  ∘ Include explicit statements about evidence base and applicability limitations. (C)
  ∘ When sex differences in effects are identified but evidence quality differs between sexes, consider separate recommendations with different grading. (C)

- Include qualifying statements in a single recommendation that note important sex or gender considerations (e.g., “This recommendation applies to all sexes; however, females may require closer monitoring due to higher risk of adverse effects”). (C)

- Acknowledge uncertainty for under‐represented populations where relevant (C), especially when:
  ∘ Evidence comes predominantly from one sex.
  ∘ Evidence base is lacking for applicability to transgender or intersex populations.
  ∘ Extrapolating findings across sex or gender groups.

To inform these structural decisions, guideline developers should consider the following:

Clinical effectiveness and safety

- Assess whether there are sex‐specific dosing requirements, contraindications or precautions. (C)
- Assess whether treatment effects, adverse effects or safety profiles differ by sex. (C)
- Consider whether long‐term hormone therapy taken by transgender people affects diagnosis, disease progression or treatment. (HI) *For example:*
  ∘ PSA levels in transgender women (male‐to‐female) may be suppressed by hormone therapy, potentially masking prostate cancer risk.
  ∘ Transgender men (female‐to‐male) receiving testosterone therapy may have altered bone density, increasing osteoporosis risk.

- Identify sex or gender differences in disease presentation, diagnostic test thresholds, disease progression, or prognosis that should inform recommendations. (C)
- Consider whether there are gender‐related differences in access to care, screening uptake, referral patterns, or access to diagnostic services that may affect how recommendations should be framed or implemented. (C)

- Consider whether there are clinical screenings or monitoring considerations specific to transgender populations that are independent of hormone therapy. (CS) *For example:*
  ∘ Cervical cancer screening remains relevant for transgender men who retain a cervix.
  ∘ Cardiovascular risk monitoring may be indicated for transgender individuals, including those not receiving hormone therapy.

- Identify any specific safety implications for pregnancy and reproductive health. (C)

Evidence quality and applicability

- Assess whether the evidence base is adequately representative of all sexes. (C)
- When evidence comes predominantly from one sex, consider whether extrapolation to the whole population is appropriate based on biological plausibility and other evidence. (C)
- Consider whether gender diverse populations are included in the evidence and consider implications for applicability. (HI)
- Evaluate whether the certainty of evidence differs between sexes, and whether it is modified by other factors such as age. (C)

Social and equity considerations in the context of unintended consequences

- Identify whether sex‐specific recommendations could inadvertently create inequitable access for specific sex or gender groups (e.g., delayed access to life‐saving treatment based on differential risk thresholds). (C)
- Assess whether recommendations reinforce harmful stereotypes or create stigma for some populations. (C)
- Consider whether it would be socially, culturally and morally appropriate to make sex‐ or gender‐specific recommendations and what the justification would be. (CS)
- Assess whether sex or gender‐based recommendations disadvantage groups in other ways (e.g., health insurance, discrimination). (C)
- Identify whether sex or gender intersects with other equity considerations (e.g., age, ethnicity, socioeconomic position, disability, sexuality) and consider how recommendations should be targeted or qualified to address compounding disadvantage ‐ for example, by specifying implementation supports or flagging populations requiring additional resources or tailored approaches. (C)

Patient values, acceptability and implementation

- Consider how sex, gender and gender identity might affect treatment acceptability, preferences or values. (C)
- Consider any cultural, religious or social barriers to implementation that differ by sex or gender. (CS)
- Are there specific consent, privacy, or confidentiality considerations, particularly in relation to gender identity and diversity. (HI)
- Identify how gender roles or responsibilities (e.g., caregiving, work patterns) might affect adherence or feasibility. (HI)
- Assess whether there are differences in healthcare‐seeking behaviours or patient‐clinician communication that should inform implementation approaches. (HI)

Resource and health system considerations

- Consider whether there are resource implications (e.g., sex‐specific monitoring requirements), delivery settings or workforce considerations that differ by sex or gender and affect implementation. (HI)
- Consider whether there are specialised services or expertise required that may not be equally accessible. (HI)

**Example:** The Spanish CPG on Gout [38] acknowledges that gendered social norms and roles shape how individuals experience and respond to the disease. For example, masculine norms may discourage men from seeking medical help or disclosing pain, while women may face stigma, lack accessible information, and experience a greater psychosocial impact on identity.

### 3.5 Step 5. Structuring the Guideline

*Core recommendation: Include a glossary of sex and gender terms and use terms consistently*

While attention to sex and gender in CPGs is improving, sex and gender terms remain inadequately used in many guidelines, and when terms are included, they may be used inconsistently or incorrectly [7, 39]. Omitted or incorrect terminology reduces the accuracy, clarity and applicability of CPG recommendations, potentially leading to confusion in clinical implementation [26]. The inclusion of a glossary of key sex and gender terms relevant to your guideline ensures clarity and minimises confusion.

Guidelines should:

- *Define key terms explicitly:* include definitions of sex and gender, and additional terms where appropriate and relevant to the guideline scope. (C)
- *Ensure accurate and consistent application of terms:* avoid using “sex” and “gender” terms interchangeably. When evidence or clinical considerations relate to both biological and social factors, specify both (e.g., sex and gender considerations in pain management). (C)
- *Specify the framework being applied*: where possible utilise a gender‐spectrum based framework [18]. If applying a binary framework, clarify why binary categories are being used and acknowledge this limitation, as well as the populations for whom the recommendations may not apply. If addressing transgender and gender diversity, clarify the terminology used and ensure consistency. (C)
- *Consider organ‐based or function‐based criteria:* in some clinical contexts, it may also be relevant to make or qualify recommendations based on the presence or absence of specific organs, anatomy or physiological functions (e.g., “people with a cervix,” and “people with a prostate”) to ensure that the health needs of transgender individuals are met. (CS)
- Note that this is an evolving and contested area of practice with differing perspectives. Terminology choices in this area involve trade‐offs and careful consideration: language intended to be inclusive of some groups may feel reductive or diminishing to others. There is no universally accepted approach. Guideline developers are strongly encouraged to consult relevant patient and public groups—including women, transgender, and non‐binary individuals—to identify preferred terminology for their specific context. When using organ‐based or function‐based language, explain the rationale clearly and ensure the approach enhances, rather than obscures clarity for both clinicians and patients. Balance precision and inclusivity with practical applicability in clinical settings. (CS)
- *Acknowledge complexity and limitations:* recognise that sex and gender are distinct but inter‐related concepts that both influence health. Acknowledge where uncertainty exists in the evidence base regarding whether observed differences are attributable to biological sex, gender‐related factors or both, and acknowledge when recommendations are based on limited evidence that does not adequately capture sex or gender diversity. (HI)

**Example:** The Australian LGBTQIA + GP Guide [40] notes use of the Australian Bureau of Statistics (ABS) Standard for Sex, Gender, Variations of Sex Characteristics and Sexual Orientation Variables [19]. This involves explicitly distinguishing between sex (biological attributes assigned at birth) and gender (social identity, lived role) in documentation and clinical interactions, ensuring clinicians use correct terminology and avoid conflating the two.

### 3.6 Step 6. Undertaking Public Consultation

*Core recommendation: Seek feedback, drawing on a range of sex and gender perspectives*

Research evidence may not fully capture the perspectives and experiences of different sex and gender populations relevant to the guide. Meaningful engagement with diverse populations is one strategy to advance equity, ensuring that guidelines reflect the values, preferences and lived experiences of all people that the guidelines are intended to serve. When undertaking public or patient consultation, ensure that diverse sex and gender perspectives are actively sought and represented. This assists in the detection of unintended consequences or disparities, improves the acceptability and feasibility of recommendations, enhances clarity and communication, and assists in addressing implementation barriers [41]. Consultation on sex and gender considerations should be embedded within broader public consultation and engagement processes that are already standard practice in guideline development, rather than as a separate activity.

Guidelines should:

- Conduct targeted consultation with different sex and gender communities to ensure guideline content is accessible and relevant to diverse populations. (HI)
- Seek explicit feedback on potential unintended consequences, barriers, or disparities affecting different sex and gender groups. (C)
- Engage people with lived experience of sexual and gender diversity to identify evidence gaps and ensure their values and preferences are reflected. (HI)

**Example:** For Australian First Nations communities, gender‐based cultural avoidance practices can impact on guideline implementation, particularly for recommendations requiring physical examination or intimate care. Cultural protocols that govern interaction between men and women may create barriers when guidelines assume mixed‐gender care delivery is universally acceptable. There is therefore a need for consultation with First Nations communities to ensure that guidelines acknowledge these cultural considerations and provide implementation options such as same‐gender healthcare providers, cultural liaison support, and flexible care delivery models that respect cultural protocols while maintaining clinical quality.

### 3.7 Step 7. Disseminating, Implementing and Evaluating the Guideline

*Core recommendation: Identify and acknowledge evidence gaps; monitor and evaluate equitable outcomes across sex and gender groups*

To support implementation, guidelines should document the evidence basis for recommendations and identify research gaps.

Guidelines should:

- Identify and acknowledge research evidence gaps pertaining to sex and gender, and provide conditional recommendations where evidence is limited or lacking for certain populations, clearly nominating these as priority areas for future research. (C)
- Monitor whether recommendations achieve equitable outcomes across populations and identify any unintended differential impacts. (C)
- Systematically review new evidence on sex and gender differences in outcomes, treatment effects, or implementations as part of scheduled guideline updates and revise recommendations accordingly. (HI)
- Provide guidance on the implications of evidence gaps and when extrapolation from other populations is reasonable and feasible. (C)

**Example:** The UK Breast Cancer guideline [42] acknowledges that almost all clinical trial evidence comes from (cisgender) women, with very limited evidence for men and trans people. By explicitly stating this, it helps clinicians apply recommendations with caution in under‐studied populations and highlights the need for new evidence.

## 4 Final Comments

The seven steps outlined here provide a framework for integrating sex and gender considerations throughout CPG development. While not every step will be equally relevant to every guideline, systematic attention to sex and gender helps ensure that recommendations are appropriate, effective, and equitable across diverse populations.

## Author Contributions

**Tania King:** investigation, funding acquisition, writing – original draft, methodology, writing – review and editing, supervision, conceptualization, validation, project administration. **Jennifer Ervin:** conceptualization, investigation, writing – original draft, methodology, writing – review and editing, validation, project administration. **Marya Yenita Sitohang:** conceptualization, investigation, writing – original draft, methodology, writing – review and editing, validation. **Kim Dalziel:** writing – original draft, methodology, writing – review and editing. **Michelle Tew:** writing – original draft, methodology, writing – review and editing. **Cara Tannenbaum:** investigation, writing – original draft, writing – review and editing, validation, methodology. **Humaira Maheen:** conceptualization, investigation, writing – original draft, methodology, writing – review and editing, validation.

## Conflicts of Interest

The authors declare no conflicts of interest.

## AI Transparency Statement

Assistive artificial intelligence (AI) tools were used to aid with initial scoping and copy editing to improve readability. The research team reviewed, edited, and verified all AI outputs to ensure accuracy and validity, and take responsibility for the final content.

## Supporting information


Supporting File

## Data Availability

Data sharing not applicable to this article as no datasets were generated or analysed during the current study.

## Footnotes

1 The current global political landscape may present ongoing challenges when conducting comprehensive evidence reviews on sex and gender in health. Recent policy shifts dismantling Diversity, Equity, and Inclusion (DEI) initiatives across US federal agencies and research institutions have created barriers to identifying and accessing sex and gender health research through funding restrictions, institutional censorship, and politicisation of gender‐related science. Guideline developers must remain vigilant to distinguish between genuine absence of evidence and politically motivated suppression of research. This requires proactive strategies including searching international databases beyond US sources, consulting experts for unpublished data, and placing greater emphasis on evidence from regions where sex and gender research continues unimpeded.

2 Note also that it is important to consider how sex and gender are recorded and defined in studies; sometimes, studies report these without explicit definition, making it difficult to draw conclusions about whether it was indeed sex or gender that was measured.
