# Supplementary material for: A Guide for the Inclusion of Sex and Gender Considerations in Clinical Practice Guidelines
Source: Clin Public Health Guidel. 2026 Sep 11;3(4):e70087. doi: 10.1002/gin2.70087 (PMC13618558; doi:10.1002/gin2.70087)
Supplement: Supplementary file 1 [file GIN2-3-e70087-s001.docx]

A Guide for the Inclusion of Sex and Gender Considerations in Clinical Practice Guidelines

Supplementary Files

Table of Contents

[Resource Files	2](#_Toc237612406)

[Case studies and examples	2](#_Toc237612407)

[Step 1: Establishing guideline development group and advisory panels	2](#_Toc237612408)

[**Recommendation**: Ensure diverse and inclusive gender representation in the guideline development group	2](#_Toc237612409)

[Step 2: Scoping the guide	2](#_Toc237612410)

[**Recommendation:** Identify sex and gender issues relevant to the guideline	2](#_Toc237612411)

[Step 3: Undertaking the evidence review	2](#_Toc237612412)

[**Recommendation:** Ensure appropriate evidence is sought, identified and considered	2](#_Toc237612413)

[Step 4: Formulating guideline recommendations	3](#_Toc237612414)

[**Recommendation:** Consider the impact of guideline recommendations on under-represented sex and gender populations	3](#_Toc237612415)

[Step 5: Structuring the guideline	3](#_Toc237612416)

[**Recommendation:** Include a glossary of sex and gender terms and use terms consistently	3](#_Toc237612417)

[Step 6: Undertaking public consultation	4](#_Toc237612418)

[**Recommendation:** Seek feedback, drawing on a range of sex and gender perspectives	4](#_Toc237612419)

[Step 7: Disseminating, implementing and evaluating the guideline	4](#_Toc237612420)

[**Recommendation:** Identify and acknowledge evidence gaps	4](#_Toc237612421)

[Checklist	5](#_Toc237612422)

[Methodology Files	6](#_Toc237612423)

[Methodological approach and overview	6](#_Toc237612424)

[Literature reviews	7](#_Toc237612425)

[Review 1: Umbrella Review	7](#_Toc237612426)

[Review 2: Synthesis of ‘Best Practice’	8](#_Toc237612427)

[Expert and lived experience consultation: Qualitative interviews	9](#_Toc237612428)

[Guide development and consultation	9](#_Toc237612429)

[References	10](#_Toc237612430)

# Resource Files

## Case studies and examples

### Step 1: Establishing guideline development group and advisory panels

### **Recommendation**: Ensure diverse and inclusive gender representation in the guideline development group

- *The European Society of Cardiology (ESC) Guideline on Cardiovascular Disease Prevention^1^ included sex and gender experts in the development team and consequently provided a more comprehensive integration of sex and gender considerations, offering clear definitions, explaining their impact on health, and differentiating risk factors, diagnostic approaches, and clinical recommendations for women and men.*

### Step 2: Scoping the guide

### **Recommendation:** Identify sex and gender issues relevant to the guideline

The following examples highlight how identifying sex and gender differences and considerations at Step 2 is fundamental to equitable healthcare. These examples were informed by expert and lived experience interviews and literature reviews conducted for this guide.

- ***Osteoporosis Screening and Management:*** *While often considered a “women's disease” men are significantly under-diagnosed and under-treated despite higher mortality rates following hip fractures. The historical focus on postmenopausal women has created gender-based barriers to men accessing preventive screening. This historical focus on postmenopausal women has created screening and treatment gaps for men. Identification of these issues could inform guideline recommendations to ensure appropriate screening thresholds, risks assessments, and treatment protocols to improve outcomes across the population.*
- ***Cardiovascular disease***: *Women often present with different symptoms to men (e.g., fatigue, nausea rather than classic chest pain), which can lead to their delayed diagnosis and under-treatment. Identification of these differences could inform guideline recommendations and lead to improved outcomes for females with cardiovascular disease.*
- ***Nephrology and Renal Transplant Care:*** *Nephrology guidelines often employ a universal approach for men and women, inadequately addressing reproductive health, contraception, and teratogenic medication risks for women of childbearing age. While specialised preconception care resources exist separately, these are not integrated within general guidelines, resulting in inconsistent discussion of contraception and pregnancy planning with female renal patients. Identification of these sex-specific considerations could inform guideline recommendations to integrate reproductive health and safety considerations into nephrology care.*

***Asthma Management in Adults:*** *Occupational exposures that trigger asthma differ by gender due to workforce segregation, with women more commonly exposed to cleaning products and healthcare-related irritants, while men face higher exposure to construction dust, industrial chemicals and agricultural agents. Some trigger identification strategies do not account for these gendered patterns of occupational exposure. Identification of these sex and gender differences could inform guideline recommendations on trigger assessment, occupational history-taking and tailored prevention strategies that reflect gendered workplace exposures.*

### Step 3: Undertaking the evidence review

### **Recommendation:** Ensure appropriate evidence is sought, identified and considered

The following are examples of considerations or steps implemented to ensure appropriate evidence is obtained.

- *Sex- and gender-specific sex terms: This Basic Search String from ‘How to Guide’ for end-users has been shown to provide higher sensitivity and specificity to sex and gender specific health literature^2^: (sex based OR sex factors OR sex distribution OR sex characteristics OR sex dimorphism OR gender difference* OR gender based) AND (gender[ti] OR sex[ti]) AND (Humans[Mesh] AND English[lang])*

*Assessing sex-specific bias: Assessments of the risk for sex-specific bias in research reports can be evaluated using the participation to prevalence ratio (PPR), a metric that links the participation of each sex to the prevalence of the disease of interest in the population. A PPR >0.8 is considered adequate or bias-free enrolment.^3^*

### Step 4: Formulating guideline recommendations

### **Recommendation:** Consider the impact of guideline recommendations on under-represented sex and gender populations

Drawn from existing clinical practice guidelines, the following are examples of the way sex and gender considerations have been incorporated into recommendations.

- *The NICE Osteoporosis guideline^4^ incorporates menopause and hypogonadism as sex-specific risk factors and uses age- and sex-differentiated thresholds (women ≥ 65 years; men ≥ 75 years) to trigger routine risk assessment.*
- *The ESC Guidelines on Cardiovascular Disease Prevention provides recommendations for cardiovascular disease assessment in specific clinical conditions, including sex-specific conditions as per the table below.^1, p.3261^*

| Clinical condition | Recommendations | Class of Recommendation | Level of Evidence |
| --- | --- | --- | --- |
| Sex-specific conditions | In women with a history of preeclampsia and/or pregnancy induced hypertension, periodic screening for hypertension and DM should be considered. | IIa | B |
|  | In women with a history of polycystic ovary syndrome or gestational DM, periodic screening for DM should be considered. | IIa | B |
|  | In women with a history of premature or stillbirth, periodic screening for hypertension and DM may be considered | IIb | B |
|  | Assessment of CVD risk should be considered in men with ED | IIa | C |
| DM - Diabetes mellitus; CVD - Cardiovascular disease; ED - Erectile dysfunction | | | |

- *The Spanish CPG on Gout^5^ acknowledges that gendered social norms and roles shape how individuals experience and respond to the disease. For example, masculine norms may discourage men from seeking medical help or disclosing pain, while women may face stigma, lack accessible information, and experience a greater psychosocial impact on identity.*
- *The Australian ADHD guideline^6^ states that:*

*“Clinicians should be aware that ADHD could be under-recognised in girls and women and that they:*

- *are less likely to be referred for assessment for ADHD*
- *may be more likely to have undiagnosed ADHD*
- *may be more likely to receive an incorrect diagnosis of another mental health or neurodevelopmental disorder, such as an anxiety or depressive disorder”*

The World Health Organisation (WHO) is developing a guideline on the health of trans and gender diverse people,^7^ which will serve as an important resource once completed.

### Step 5: Structuring the guideline

### **Recommendation:** Include a glossary of sex and gender terms and use terms consistently

Drawn from existing CPGs, the following are examples of the way key terms have been defined within clinical practice guidelines.

- *The Australian LGBTQIA+ GP Guide^8^ notes use of the Australian Bureau of Statistics (ABS) Standard for Sex, Gender, Variations of Sex Characteristics and Sexual Orientation Variables.^9^ This involves explicitly distinguishing between sex (biological attributes assigned at birth) and gender (social identity, lived role) in documentation and clinical interactions, ensuring clinicians use correct terminology and avoid conflating the two.*
- *The European CVD Prevention CPG^1^ explicitly defines sex and gender as per the World Health Organisation*.

*"There is, at present, no official ESC position on the specific terminology to be used. According to the World Health Organization (WHO), sex ‘refers to the different biological and physiological characteristics of females, males, and intersex persons, such as chromosomes, hormones and reproductive organs’. This is to be distinguished from gender, which ‘refers to the characteristics of women, men, girls and boys that are socially constructed. This includes norms, behaviours and roles associated with being a woman, man, girl or boy, as well as relationships with each other. As a social construct, gender varies from society to society and can change over time’. The Global Health 50/50 definition further states that gender refers ‘to the socially constructed norms that impose and determine roles, relationships, and positional power for all people across their lifetime.’”^1^*

### Step 6: Undertaking public consultation

### **Recommendation:** Seek feedback, drawing on a range of sex and gender perspectives

The following examples highlight how public consultation is needed to aid in the identification of sex and gender specific considerations in CPG language, approaches and outcomes considerations. These examples were informed by expert and lived experience interviews and literature reviews conducted during the development of this guide.

- ***Healthcare Service Delivery for Indigenous and Torres Strait Islander Communities:*** *For First Nations communities, gender-based cultural avoidance practices can impact on guideline implementation, particularly for recommendations requiring physical examination or intimate care. Cultural protocols that govern interaction between men and women may create barriers when guidelines assume mixed-gender care delivery is universally acceptable. There is therefore a need for consultation with First Nations communities to ensure that guidelines acknowledge these cultural considerations and provide implementation options such as same-gender healthcare providers, cultural liaison support, and flexible care delivery models that respect cultural protocols while maintaining clinical quality.*
- ***Cervical Screening:*** *Screening programs that target “women aged 25-74" may exclude transgender men with a cervix from recall systems and create barriers to accessing services, while terminology choices can affect how different populations experience guidelines. Consultation with transgender communities and women's health advocates is essential to: define eligibility based on anatomy rather than gender identity; ensure health systems can identify all eligible individuals; navigate language considerations; and provide guidance on inclusive service delivery. Such consultation is key to ensuring that screening guidelines promote equitable access for all who require cervical screening.*
- ***Eating Disorder Treatment:*** *Guidelines for eating disorders have typically focused almost exclusively on experiences of adolescent girls and young women, with recommendations often applying female-centred assessment (e.g., focus on menstruation, breast development concerns). This fails to acknowledge males and gender diverse populations experiencing eating disorders, contributing to invisibility, stigma and delayed diagnosis. Consultation with males and gender diverse populations is central to ensuring gender-inclusive assessment approaches and specialised referral pathways.*

### Step 7: Disseminating, implementing and evaluating the guideline

### **Recommendation:** Identify and acknowledge evidence gaps

Drawn from existing guidelines, the following are examples of the way evidence gaps have been identified and acknowledged within CPGs.

- *The UK Breast Cancer guideline^10^ acknowledges that almost all clinical trial evidence comes from (cisgender) women, with very limited evidence for men and trans people. By explicitly stating this, it helps clinicians apply recommendations with caution in under-studied populations and highlights the need for new evidence.*
- *The Canadian Cardiovascular Society’s guidelines on the Acute Management of ST-Elevation Myocardial Infarction^11^ provide an example of how CPGs can transparently report and advise their readers when decisions for female patients (for example) are unable to be supported by existing evidence.*

*“In addition, we systematically appraised the included literature for modifying effects of sex and gender on outcomes and determined whether the quality of evidence and strength of recommendations should differ on the basis of sex or gender. Although the systematic appraisal of sex and gender considerations of included literature as part of clinical practice guideline development was feasible, inadequate enrolment of women in randomized trials, lack of publication of main outcomes stratified according to sex, and lack of inclusion of gender as a study variable prevented us from providing sex- and gender-specific strengths of recommendations for the clinical questions evaluated. Although we make the agnostic assumption that the recommendations in this guideline hold equally for men and for women, we acknowledge that the published literature are inadequate to clearly and objectively confirm this.” ^11^*

- *The Australian ADHD guideline^6^ states that “Research gaps include: … an understanding of the impacts of the menstrual cycle, pregnancy, birth, breast feeding and menopause on girls and women with ADHD including optimal treatment.”*

## Checklist

To support feasibility, recommendations are categorized as: **Core** (**C**, minimum requirements for all guidelines); **High-Impact** (**HI** priority where resources permit); or **Context-Specific** (**CS**, recommended where relevant to the condition, population, or guideline scope).

| **Step** | **Consideration/**  **Recommendation** | **Prompts** |
| --- | --- | --- |
| **1: Establishing guidelines development group** | - Ensure diverse and inclusive gender representation in the guideline development group | - Ensure equitable representation of women on development and advisory panels (C) - Ensure representation across gender diversity where applicable (CS) - Include multidisciplinary expertise in sex and gender health (C) |
| **2: Scoping the guideline** | - Identify sex and gender issues relevant to the guideline | - Conduct scoping to identify known and potential sex or gender differences.   Consider epidemiology, biological mechanisms, access and presentation, diagnosis and treatment, evidence gaps, implementation factors, and equity implications (C)   - Ensure research questions explicitly include sex and gender considerations (C) - Define and specify sex- and gender-disaggregated terms a priori (C) - Consult with diverse sex and gender communities (CS) |
| **3: Undertaking the evidence review** | - Ensure appropriate evidence is sought, identified and considered. | - Include sex and gender-specific search terms, including single-sex or single-gender studies in evidence reviews where appropriate (C) - Record whether sex or gender analyses and results are presented; note the sex or gender distribution of study populations (C) - When conducting subgroup analyses, assess whether analyses were pre-specified in original studies; if post-hoc, frame as exploratory and hypothesis-generating (C) - Evidence synthesis: prioritise sex-and gender-disaggregated evidence; avoid over-interpretation of data; consider publication bias; consult biostatistician or expert opinion when uncertain (C) - Evidence appraisal: evaluate whether the evidence base represents and is applicable to sex and gender groups (C) - Evidence to decision frameworks: assess whether benefits, harms, feasibility, baseline risk, and social values differ by sex or gender; consider differential application due to bias; assess whether recommendations reflect identified differences (C) - Ensure transparent reporting, particularly when there is insufficient data from one sex or gender, but recommendations apply to the whole population (C) |
| **4: Formulating guideline recommendations** | - Consider the impact of guideline recommendations on under-represented sex and gender populations. | - Provide separate recommendations for males and females (as a minimum) when evidence shows clinically meaningful differences in treatment effects, safety profiles or outcomes (C) - Assess sex-specific dosing, contraindications, precautions, treatment effects, adverse effects and safety profiles (C), - Identify gender-related contraindications and precautions (C) - Assess whether the evidence base adequately represents all sexes and gender diverse populations (C) - Where evidence arises mainly from one sex, consider appropriateness of extrapolating based on biological plausibility(C) - Assess hormone therapy interactions where relevant (CS) - Address pregnancy and reproductive health implications (C) - Consider implications for intersex and gender diverse individuals where appropriate (CS) - Identify potential for unintended consequences of sex or gender-specific recommendations in terms of access, stigma, discrimination or harm (C) - Acknowledge intersectionality and consider the way sex and gender interact with other factors (such as age, ethnicity, socioeconomic position, disability, sexuality) and consider approaches to reduce compounding disadvantage(C) - Consider how sex, gender and gender identity might affect treatment acceptability, preferences or implementation (C) - Acknowledge uncertainty for under-represented populations when evidence base lacking or mainly from one sex (C) |
| **5: Structuring the guideline** | - Include a glossary of sex and gender terms and use terms consistently. | - Define sex and gender terms relevant to the guideline (C) - Ensure accurate and consistent application of sex and gender terms (C) - Specify the sex and gender frameworks being applied (C) - Consider and justify use of organ-based or function-based criteria where clinically relevant; consult relevant public and lived experience experts on terminology preferences (CS) - Acknowledge complexity and limitations of concepts being used (HI) |
| **6: Undertaking public consultation** | - Seek feedback, drawing on a range of sex and gender perspectives | - Conduct targeted consultation with different sex and gender groups to ensure accessibility and relevance of guideline content (HI) - Seek feedback on potential unintended consequences, barriers or disparities affecting different sex and gender groups(C) - Engage consumers with lived experience to identify evidence gaps and ensure diverse values and preferences are reflected (HI) |
| **7: Disseminating, implementing and evaluating the guideline** | - Identify and acknowledge evidence gaps. | - Identify and acknowledge evidence gaps; provide conditional recommendations when evidence is limited and identify areas where research is needed (C) - Monitor impact of recommendations including unintended outcomes (C) - Review and update CPGs as new evidence becomes available (HI) - Provide guidance on implications of evidence gaps and when extrapolation from other populations is reasonable (C) |

# Methodology Files

## Methodological approach and overview

This guide was developed through a multi-component methodology comprising expert and lived experience consultation, participatory research, and two literature reviews. Detailed methodology for the literature reviews and interviews are provided in the subsections below.

Guide development drew on findings from these reviews and interview findings alongside established frameworks for health equity in guideline development, including the GIN-McMaster Guideline Development health equity considerations,^12^ the Cochrane Equity Methods Group (PROGRESS-Plus),^13,14^ the Grading of Recommendations Assessment, Development and Evaluation (GRADE) equity guidelines,^15^ and the Sex and Gender Equity in Research (SAGER) guidelines.^16,17^ Australian guidelines were also used, including the National Health and Medical Research Council (NHMRC)’s Guideline Standards, ^18^ Guidelines for Guidelines: Equity,^19^ and Statement on Gender, Variations of Sex Characteristics and Sexual Orientation in Health and Medical Research.^20^

Clinical expert advisory expertise was embedded across the project, drawing on the existing group of experts already available through Melbourne Health Technology and Value Assessment Collaboration (M-VAC). Clinical experts from M-VAC provided guidance on project design, assisted with recruitment for qualitative interviews, and provided feedback on the draft guide.

In line with best practice participatory research approaches, we employed three patients/consumers with lived experience (of either gender/sexual diversity; sex-specific health condition) as consultants across the project. Lived experience participants were involved in developing the project plan, participated in qualitative interviews, and reviewed and provided feedback on the draft guide.

Ethics approval was obtained from the University of Melbourne’s Human Research Ethics Committee (#2025-33040-68447-3).

## Literature reviews

To examine and assess the extent of existing research and recommendations for the inclusion of sex and gender in clinical guidelines, we conducted two international literature reviews: an umbrella review synthesising existing research on the inclusion of sex and gender in clinical practice guidelines, and a targeted analysis of best-practice guidelines identified through that review. Methodological details are as follows.

### Review 1: Umbrella Review

- This review scoped and synthesised the international research on the inclusion of sex and gender in CPGs. Conducted as an umbrella review, it focused on existing reviews and analyses of clinical practice guidelines (CPGs) focused on sex and gender.
- The review protocol was prospectively registered in Prospero (CRD420251117265) and the PRIOR (Preferred Reporting Items for Overviews of Reviews) statement in 2022 was adhered to in summarising the current global evidence on sex and gender considerations in clinical guideline practices.
- An umbrella review design from the Joanna Briggs Institute (JBI)^21^ was used, with inclusion of high quality narrative or general reviews to provide a higher-level summary of sex and gender considerations in CPGs. While the JBI traditionally focuses on systematic reviews, we included other types of reviews because research on sex and gender inclusion in CPGs remains limited and often not labelled or indexed as systematic reviews. This broader inclusion allowed a more comprehensive synthesis of available evidence.
- We defined and tested our search strategy in consultation with University of Melbourne librarians before conducting a comprehensive search in multiple databases.
  - Literature searches were conducted between 2025 to 09 July 2025 in eight databases: Medline (via OVID), Scopus, Embase (via OVID), CINAHL (via EBSCOhost), Sociological Abstracts (via ProQuest), The Cochrane Database of Systematic Reviews (via OVID), JBI Evidence Synthesis (via OVID), Database of Abstracts of Reviews of Effects (via OVID) due to their relevance to the topic of this study interest. Search alerts were also set for each database, and after the initial comprehensive screening, ongoing screening of search alerts continued until November 14th, 2025.
  - The search was conducted by applying a three-tiered search strategy, combining terms for clinical practice guidelines, sex and gender concepts, and review types (see Table S1). The search strategy was first developed in Medline, peer-reviewed by the research team, and then employed across all databases. The search was limited to published reviews in English and publication after the year of 2000, but no geographical restriction was applied.
  - As best practice for reviews of this kind, reference lists of included studies were scanned for further possible articles, and we set up “search alerts” to ensure that we captured any further studies that arise since the conduct of the review (in June/July). These were continuously screened post initial search through till November 14^th^, 2025.

#### Table S1: Tiered search strategy

| Tier 1 | Clinical practice guidelines | "clinical practice guideline*", "practice guideline*", "clinical guideline*".ti (*restrict to title only)* |
| --- | --- | --- |
| Tier 2 | Sex and gender | "sex factors", "sex characteristics", "gender identity", "transgender persons", "women's health", "men's health", sex, gender, (sex adj3 (difference* OR specific OR based OR factor* OR consideration*)), (gender adj3 (difference* OR specific OR based OR factor* OR consideration*)), (sex adj3 (disaggregat* OR sensitiv* OR integrat* OR inclusion OR representation )) OR (gender adj3 (disaggregat* OR sensitiv* OR integrat* OR inclusion OR representation)), women*, female*, men, male*, transgender, "non-binary", "gender-diverse", "gender diverse", LGBTI*, LGBTQ*.mp *(can be found anywhere)* |
| Tier 3 | Review types | "systematic review*", "meta-analysis", "scoping review*", "literature review*”, "narrative review*”,ti,ab. *(restrict to title and abstract only)* |

- Records identified from the search results were imported into Covidence,^22^ a web-based tool for conducting reviews. Potential review articles for inclusion (title/abstract and then full text) were independently screened by two reviewers, followed by data extraction - also independently conducted by two researchers. Discrepancies during the screening process were resolved by two reviewers through discussion and by discussions with a third reviewer if consensus was not achieved. Following consensus, data was consolidated into a final data extraction form.
- Quality assessment of the included reviews was independently assessed by two researchers using the JBI Critical Appraisal Checklist for Systematic Reviews and Research Syntheses.^21^
- A descriptive summary was firstly performed to present the review type, included CPGs characteristics, and sex and gender considerations in the CPGs, followed by a narrative synthesis for both quantitative and qualitative findings to summarise how sex and gender considerations were integrated across the included reviews, identifying common issues and recommendations.
- We note that this review titled “Inclusion of Sex and Gender in Clinical Practice Guidelines: An Umbrella Review” is currently in press .^23^

### Review 2: Synthesis of ‘Best Practice’

- This review comprised a targeted analysis of CPGs identified as 'best practice' with respect to their consideration of sex and gender, drawing directly from CPGs highlighted within the included reviews of the umbrella review (Review 1).
- Rather than conducting an independent database search, a targeted approach was adopted: two reviewers independently identified CPGs that authors in Review 1 had highlighted as addressing sex and gender more comprehensively than the broader sample.
- From the seven reviews^24-30^ included in Review 1, 14 best-practice CPGs were identified across five reviews, spanning multiple countries and clinical conditions. These guidelines were retrieved for analysis.
- Data extraction was conducted by one researcher and cross-checked by a second reviewer. Data captured included: guideline source, clinical scope, key strengths regarding sex and gender consideration, and identified gaps.
- Findings were synthesised narratively and cross-analysed to identify patterns, consistencies, and limitations across the best-practice sample.
- Across the 14 ‘best practice’ CPGs reviewed, biological sex differences were consistently recognised and increasingly integrated into risk assessment, diagnostic thresholds, and treatment guidance. However, translation of gender identity, social roles, and behavioural determinants into actionable recommendations remained limited. Inclusivity for transgender and non-binary people was largely absent, and guidelines did not appear to systematically integrate gendered determinants or equity frameworks into actionable care pathways. Overall, whilst encouraging progress was identified, this review identified considerable opportunity to enhance consistency and extend best practice to CPGs more broadly.

## Expert and lived experience consultation: Qualitative interviews

- In-depth qualitative interviews were conducted with 31 participants including clinicians, academics, guideline experts, NHMRC stakeholders, and individuals with lived experience of sex and gender in the healthcare system. These individuals represented diverse gender identities, including men, women, transgender persons, non-binary persons, and people of varying sexual orientations. The populations and areas of expertise represented are detailed in Table S2 below.
- To ensure a breadth of perspectives, recruitment sought participation across diverse backgrounds and intersectional positions, including gender and sexually diverse and transgender populations (both clinicians and those with lived experience), First Nations peoples, culturally and linguistically diverse (CALD) populations, and people with disability.
- Recruitment drew on existing partner organisations and M-VAC to identify potential participants, facilitate recruitment, and ensure broad representation of expertise, experience, and perspectives. This was supplemented by a snowballing approach, whereby participants suggested and facilitated the recruitment of others.
- Participants who agreed to participate were provided with a plain language statement prior to interview, documenting relevant project information. Verbal and written consent was obtained before each interview commenced.
- Interviews were conducted online via Zoom, each approximately 30 minutes in length, and were audio-recorded with participants' consent. Recordings were transcribed using Zoom transcription tools.
- Transcribed data was imported into NVivo and analysed using thematic analysis,^31,32^ with coding frameworks developed iteratively to synthesise findings across the interview sample.
- We note that a publication resulting from these findings is pending titled **“Perspectives on Sex and Gender Inclusion in Clinical Practice Guidelines: a qualitative study”** (currently under review).

Table S2: Interview participant characteristics

| Populations covered by interviewees included: | Areas of expertise covered by interviewees included: | |
| --- | --- | --- |
| Children/adolescents | Sexual health | Psychology |
| Men | Cardiology | Men’s health (general) |
| Women | Gastroenterology | Women’s health (general) |
| LGBTIQ+ populations | Orthopaedics | LGBTIQ+ health |
| Trans populations | Endocrinology | Guideline expertise |
| CULTURALLY and Linguistically diverse (CALD) populations | Nephrology | Health and social equity |
| First nations | Obstetrics | Health policy |
| People with disability | Oncology | Health systems |
|  | Health research funding |  |

## Guide development and consultation

- Findings from the literature reviews and interviews were synthesised alongside established frameworks for guideline development and health equity to inform development of a draft guide.
- Feedback on the draft was obtained through a staged consultation process. Initial consultation with the NHMRC focused on guide structure and alignment with NHMRC processes. A second phase sought feedback from the Department, clinicians across diverse fields, an international expert on sex and gender, and CPG developers, with a focus on suitability, applicability, and actionability. In the final phase, lived experience consultants reviewed the draft to ensure inclusive and appropriate sex and gender language throughout.
- The guide was refined following each round of feedback, with a final review conducted by the NHMRC and the Department prior to completion.

# References

1.	Visseren FLJ, Mach F, Smulders YM, et al. 2021 ESC Guidelines on cardiovascular disease prevention in clinical practice. *Eur Heart J* 2021; **42**(34): 3227–337.

2.	Song MM, Simonsen CK, Wilson JD, Jenkins MR. Development of a PubMed Based Search Tool for Identifying Sex and Gender Specific Health Literature. *J Womens Health (Larchmt)* 2016; **25**(2): 181–7.

3.	Tannenbaum C, Norris CM, McMurtry MS. Sex-Specific Considerations in Guidelines Generation and Application. *Can J Cardiol* 2019; **35**(5): 598–605.

4.	National Institute for Health and Care Excellence (NICE). Osteoporosis: assessing the risk of fragility fracture, 2017.

5.	GuipClinGot working group. Clinical Practice Guidelines for the Management of Patients with

Gout, Madrid, Spanish Society of Rheumatology (SER), 2020. Madrid, Spain: Spanish Society of Rheumatology (SER), 2020.

6.	Australian ADHD Guideline Development Group. Australian evidence-based clinical practice guideline for Attention Deficit Hyperactivity. Melbourne, Australia: Australasian ADHD Professionals Association, 2022.

7.	World Health Organisation. Update on the Guideline Development Group on the health of trans and gender diverse people. 2024. <https://www.who.int/news/item/20-06-2024-update-on-the-guideline-development-group-on-the-health-of-trans-and-gender-diverse-people>.

8.	General Practice Supervisors Australia (GPSA). Guide for providing care to LGBTQIA+ patients in general practice. Bendigo: GPSA; 2023. Available from:. Bendigo, Victoria: GPSA, 2023.

9.	Australian Bureau of Statistics (ABS). Standard for Sex, Gender, Variations of Sex Characteristics and Sexual Orientation Variables. Canberra: ABS, 2020.

10.	National Institute for Health and Care Excellence (NICE). Early and locally advanced breast cancer: diagnosis and management (NG101). London, UK: NICE, 2018.

11.	Wong GC, Welsford M, Ainsworth C, et al. 2019 Canadian Cardiovascular Society/Canadian Association of Interventional Cardiology Guidelines on the Acute Management of ST-Elevation Myocardial Infarction: Focused Update on Regionalization and Reperfusion. *Can J Cardiol* 2019; **35**(2): 107–32.

12.	Dewidar O, Sayfi S, Pardo JP, et al. Enhancing health equity considerations in guidelines: health equity extension of the GIN-McMaster Guideline Development Checklist. *EClinicalMedicine* 2025; **82**: 103135.

13.	O'Neill J, Tabish H, Welch V, et al. Applying an equity lens to interventions: using PROGRESS ensures consideration of socially stratifying factors to illuminate inequities in health. *J Clin Epidemiol* 2014; **67**(1): 56–64.

14.	Welch VA PJ, Jull J, Hartling L, Klassen T, Kristjansson E, Pardo Pardo J, Petticrew M, Stott DJ, Thomson D, Ueffing E, Williams K, Young C, Tugwell P. . Chapter 16: Equity and specific populations [last updated October 2019]. In: Higgins JPT TJ, Chandler J, Cumpston M, Li T, Page MJ, Welch VA (editors). , editor. Cochrane Handbook for Systematic Reviews of Interventions version 65 Cochrane, 2024 Available from cochraneorg/handbook. Cochrane; 2024.

15.	Akl EA, Welch V, Pottie K, et al. GRADE equity guidelines 2: considering health equity in GRADE guideline development: equity extension of the guideline development checklist. *J Clin Epidemiol* 2017; **90**: 68–75.

16.	Heidari S, Babor TF, De Castro P, Tort S, Curno M. Sex and Gender Equity in Research: rationale for the SAGER guidelines and recommended use. *Res Integr Peer Rev* 2016; **1**: 2.

17.	Van Epps H, Astudillo O, Del Pozo Martin Y, Marsh J. The Sex and Gender Equity in Research (SAGER) guidelines: Implementation and checklist development. *European Science Editing* 2022; **48**: e86910.

18.	The National Health and Medical Research Council (NHMRC). Procedures and Requirements for Meeting the NHMRC Standards for Clinical Practice Guidelines, Version 2.0. 2025. <https://www.nhmrc.gov.au/about-us/publications/procedures-and-requirements-meeting-nhmrc-standards-clinical-practice-guidelines>.

19.	The National Health and Medical Research Council (NHMRC). Guidelines for Guidelines: Equity. 2018.

20.	The National Health and Medical Research Council (NHMRC) and the Department of Health and Aged Care. Statement on Sex, Gender, Variations of Sex Characteristics and Sexual Orientation in Health and Medical Research. 2024. <https://www.nhmrc.gov.au/research-policy/gender-equity/statement-sex-and-gender-health-and-medical-research#block-views-block-file-attachments-content-block-12025>).

21.	Aromataris E, Fernandez R, Godfrey C, Holly C, Khalil H, Tungpunkom P. Umbrella Reviews (2020). In: Aromataris E LC, Porritt K, Pilla B, Jordan Z,, ed. JBI Manual for Evidence Synthesis; 2024.

22.	Veritas Health Innovation. Covidence systematic review software. Melbourne, Australia: Covidence; 2023.

23.	Sitohang. MY, Ervin. J, Maheen. H, Dalziel. K, King. T. Inclusion of Sex and Gender in Clinical Practice Guidelines: An Umbrella Review. *BMJ Open Quality* 2026; **In Press**.

24.	Gonzalez Ramos AM. Do clinical practice guidelines incorporate sex and gender evidence? *Womens Health (Lond)* 2024; **20**: 17455057241302993.

25.	Hulme R, Wilkinson O, Haythornthwaite E, et al. Are sex and gender dimensions accounted for in NICE guidelines? A systematic review of 223 clinical guidelines. *BMJ Public Health* 2025; **3**(2): e002510.

26.	Kirkman M, Honda T, McDonald SJ, et al. Consideration of sex and gender: an analysis of Australian clinical guidelines. *Med J Aust* 2025; **222**(4): 205–9.

27.	Tannenbaum C, Clow B, Haworth-Brockman M, Voss P. Sex and gender considerations in Canadian clinical practice guidelines: a systematic review. *CMAJ Open* 2017; **5**(1): E66–E73.

28.	Naghipour A, Becher E, Gemander M, Oertelt-Prigione S. Designing clinical practice guidelines for equitable, inclusive, and contextualised care. *BMJ* 2025; **391**: e085684.

29.	Rathbone T, Truong C, Haldenby H, et al. Sex and gender considerations in low back pain clinical practice guidelines: a scoping review. *BMJ Open Sport Exerc Med* 2020; **6**(1): e000972.

30.	Keuken DG, Haafkens JA, Moerman CJ, Klazinga NS, ter Riet G. Attention to sex-related factors in the development of clinical practice guidelines. *J Womens Health (Larchmt)* 2007; **16**(1): 82–92.

31.	Parkinson S, Eatough V, Holmes J, Stapley E, Midgley N. Framework analysis: a worked example of a study exploring young people’s experiences of depression. *Qualitative research in psychology* 2016; **13**(2): 109–29.

32.	Ritchie J, Spencer L. Qualitative data analysis for applied policy research. Analyzing qualitative data: routledge; 2002: 173–94.
